# How do women at increased breast cancer risk perceive and decide between risks of cancer and risk‐reducing treatments? A synthesis of qualitative research

**DOI:** 10.1002/pon.4349

**Published:** 2017-01-26

**Authors:** Hannah G. Fielden, Stephen L. Brown, Pooja Saini, Helen Beesley, Peter Salmon

**Affiliations:** ^1^ Department of Psychological Sciences University of Liverpool Liverpool UK; ^2^ CLAHRC North West University of Liverpool Liverpool UK

**Keywords:** breast cancer, meta‐synthesis, qualitative research, risk perception, risk reduction

## Abstract

**Objective:**

Risk‐reducing procedures can be offered to people at increased cancer risk, but many procedures can have iatrogenic effects. People therefore need to weigh risks associated with both cancer and the risk‐reduction procedure in their decisions. By reviewing relevant literature on breast cancer (BC) risk reduction, we aimed to understand how women at relatively high risk of BC perceive their risk and how their risk perceptions influence their decisions about risk reduction.

**Methods:**

Synthesis of 15 qualitative studies obtained from systematic searches of SCOPUS, Web of Knowledge, PsychINFO, and Medline electronic databases (inception‐June 2015).

**Results:**

Women did not think about risk probabilistically. Instead, they allocated themselves to broad risk categories, typically influenced by their own or familial experiences of BC. In deciding about risk‐reduction procedures, some women reported weighing the risks and benefits, but papers did not describe how they did so. For many women, however, an overriding wish to reduce intense worry about BC led them to choose aggressive risk‐reducing procedures without such deliberation.

**Conclusions:**

Reasoning that categorisation is a fundamental aspect of risk perception, we argue that patients can be encouraged to develop more nuanced and accurate categorisations of their own risk through their interactions with clinicians. Empirically‐based ethical reflection is required to determine whether and when it is appropriate to provide risk‐reduction procedures to alleviate worry.

## 1 BACKGROUND

The development of technologies to identify and protect individuals at high risk of cancer or its recurrence is an area of continuing medical advance.^1^, ^2^, ^3^, ^4^ However, risk‐reducing procedures are often invasive and carry iatrogenic risk. For patients to make informed decisions about risk reduction, they therefore should understand and weigh risks of disease and the long and short‐term risks and benefits associated with different treatments.^5^

Breast cancer (BC) risk reduction focuses the need to weigh risks and benefits acutely, because informed estimates of BC risk are available and risk‐reduction treatments are effective but carry risks.^6^ Women with high BC risk can be identified from family history, with the latter often mediated by identified gene mutations.^5^ Mutations in BRCA 1 or 2 genes and a history of affected first‐degree relatives confer an 80% to 90% lifetime risk, whilst mutations of other genes carry lower risks.^6^ Ashkenazi, Icelandic, Swedish, Hungarian, and French‐Canadian populations have higher rates of mutations.^7^ Previous BC also increases a woman's risk of developing a new BC.^7^ Several risk‐reduction procedures are available, with well‐understood risks and benefits. Screening, using radiographic mammography, ultrasound, or magnetic resonance imaging,^8^ cannot prevent cancer but enables early detection, which improves prognosis. However, screening can miss cancers, and false positive results cause unnecessary alarm.^9^ Chemoprevention, through selective estrogen receptor modulators or aromatase inhibitors,^10^, ^11^ can reduce incidence of estrogen receptor positive and negative cancer in postmenopausal women.^12^ Selective estrogen receptor modulators increase risk of endometrial cancer, thromboembolic events and menstrual and skin complaints, and aromatase inhibitors may reduce bone growth and contribute to infertility and liver and kidney dysfunction.^13^ Risk‐reducing mastectomy (RRM) can improve life expectancy in women with BRCA mutations. In lower risk BC survivors, RRM may reduce risk of new BC but has not been shown to improve life expectancy because it cannot reduce the likelihood of metastatic disease.^14^ Risk‐reducing mastectomy is irreversible, carries surgical risks, may require follow‐up surgery, and can cause physical discomfort and emotional distress linked to breast appearance and feelings of damaged femininity.^15^, ^16^ Bilateral salpingo‐oophorectomy (surgical removal of the ovaries and fallopian tubes) can reduce the risk of BC by up to 50% and risk of ovarian cancer by 90% to 95 %, although the extent of BC risk reduction has recently been questioned.^17^ Oophorectomy is irreversible, risks surgical complications, and causes infertility and premature menopause.^17^, ^18^ Therefore, women at high risk of BC face complex choices about risk mitigation. Practitioners caring for women face corresponding challenges around how to help women weigh risks and benefits in making risk‐reducing decisions.

According to early “likelihood‐value” theories of decision making, people should weigh risks and benefits of different decision options by estimating the likelihood and personal value of potential outcomes and select the option that offers the optimal combination of these.^19^, ^20^ However, people do not, in practice, report thinking about risk in continuous estimates of likelihood and value^21^, ^22^ and measures of these poorly predict behaviour.^23^, ^24^ The evidence suggests, instead, that people base risk perceptions and associated decisions on mental heuristics, ie, “rules of thumb” or approximations that allow easier decision making.^24^ Examples are the “affect heuristic,” whereby people's inferences of risk are guided by their emotional feelings, and the “availability heuristic,” whereby people infer risk from the ease with which risk‐related information can be recalled.^25^

Whilst heuristics can reduce the extent to which people contemplate objective risks and logically integrate them into decision making, they might also improve decisions by allowing people to respond to complex information that they would otherwise be unable to assimilate in a more rational way.^26^ A large and diverse range of heuristics have been documented, but many are specific to the demands and contexts of particular decisions.^27^ Thus, women's risk perception and decision making in BC risk reduction cannot be understood through merely appreciating that they use heuristics but requires detailed understanding of the heuristics they use and the influence of these on decision making. We are aware of no work that reviews this evidence in BC risk reduction, and this was the aim of the present study. The measurement procedures of quantitative research in this context necessarily presuppose the main ways in which risk perception and decision making vary. Consistent with our inductive aim, we therefore focused on qualitative research. Our specific aims were, first, to synthesise qualitative literature concerning how women with elevated risk for BC perceive risk and how these perceptions influence decision making about risk reduction and, second, to draw implications for how clinicians can help women make these decisions.

## 2 METHODS

### 2.1 Inclusion and exclusion criteria

We included peer‐reviewed qualitative studies that examined risk perception or decision making in adult women at high risk for BC. We used a broad definition of risk perception as “an individual's personal understandings of BC risk and of the risks and benefits of risk‐reduction options.” Higher risk groups included women with established genetic mutations (eg, BRCA 1/2), or familial risk factors (affected first‐degree relatives), higher scorers on predictive scales derived from epidemiologic analyses of risk factors, members of ethnic populations characterized by higher risk, and women who had previously been examined with BC. Studies of women currently under treatment for BC were excluded. Inclusion was limited to English language reports. Databases (see below) were searched from inception to June 2015.

### 2.2 Search strategy

Search terms and alternatives were initially identified using several reviews relevant to this area^22^, ^23^ and then augmented by scoping searches. Terms in the title, abstract, or keywords relevant to BC (“breast cancer” or “breast carcinoma” or “breast neoplasm”) and risk perception (“risk perception” or “risk understanding” or “perceived risk” or worry or dread or “anticipated emotion” or “anticipatory emotion” or emotion* or vulnerability) and qualitative methodology (qualitative or “Grounded theory” or IPA or “interpretative phenomenological analysis” or “thematic analysis” or “content analysis” or “narrative Analysis” or “conversation analysis” or “discourse analysis” or interview* or “focus groups”) were searched.

The electronic databases PsycINFO (1879‐2015), Medline (1948‐2015), Web of Knowledge (1900‐2015), and Scopus (1960‐2015) were searched. Reference lists from previous systematic reviews were also searched. Searches were combined, and duplicates removed before study selection. Hand searches were also conducted of the reference lists of the included articles.

### 2.3 Study selection

**Figure 1:**
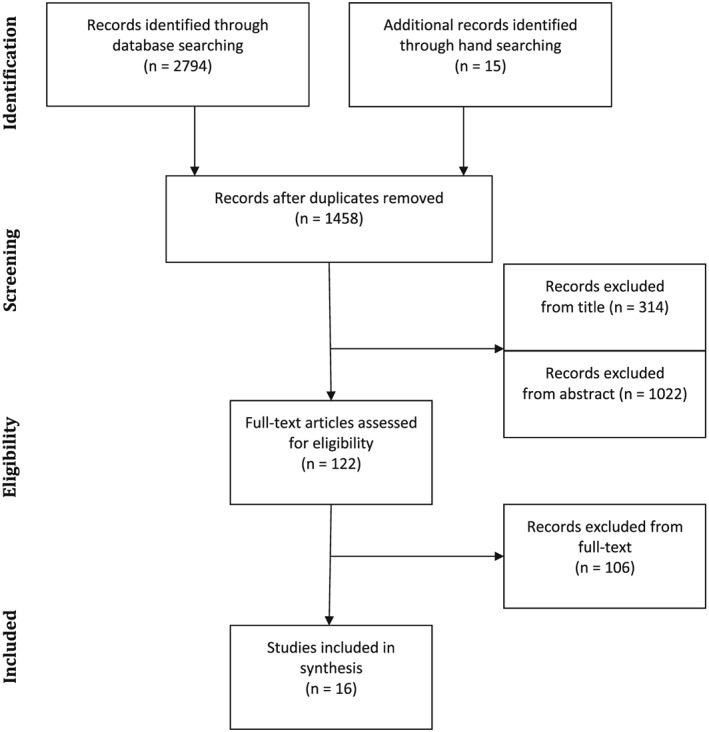
Preferred Reporting Items for Systematic reviews and Meta‐Analysis flow diagram of article selection process

Using the electronic databases, search terms were identified from titles, abstracts, and keywords. Following the search, identified studies were assessed for inclusion. Initially, HGF screened all identified titles and then the abstracts of selected titles for potential inclusion. Then all papers identified as potentially relevant were read by HGF who assessed whether they met the inclusion criteria. When this was unclear, SLB also read the study and a joint decision was reached. Stage of exclusion and the reasons for exclusion were recorded (Table 1). Figure 1 describes study selection using the Preferred Reporting Items for Systematic reviews and Meta‐Analysis^28^ flow diagram.

**Table 1:** Reasons for exclusion of studies by stage of selection

	Title Screening	Abstract Screening	Full Text Screening
Sample (eg, not a high risk sample)	282	380	29
Topic (eg, not about risk perception)	0	423	57
Method (eg, not qualitative)	3	183	6
Study type (eg, not primary data—review or commentary)	1	27	12
Duplicate not previously identified	28	9	2

### 2.4 Data synthesis

We wanted to develop new theoretical insights, grounded in the findings of individual studies but with general applicability across those studies and, therefore, potentially beyond them. Thus, we took an inductive approach, drawing upon grounded formal theory.^29^, ^30^ This approach starts with a descriptive analysis of data from the reviewed studies (including the research question, sample description, inductive categories arising from the authors' analysis, and the illustrative data contained in the reports) but then progresses to a theoretical analysis. The key method of analysis is constant comparison within and across studies to detect convergences and resolve inconsistencies. Our synthesis was at the level of reported findings rather than the authors' interpretations, and we sometimes drew different theoretical conclusions from the authors in the context of the developing analysis. HGF peformed a preliminary synthesis, first developing a descriptive analysis then a theoretical integration of this analysis. Then HGF and SLB interrogated this to identify consistencies and inconsistencies with the source data, and SLB provided a reformulated model. SLB and HGF then compared the reformulated model to the preliminary synthesis. The final synthesis arose through discussion amongst all authors.

## 3 RESULTS

Table 2 summarises^31^, ^32^, ^33^, ^34^, ^35^, ^36^, ^37^, ^38^, ^39^, ^40^, ^41^, ^42^, ^43^, ^44^, ^45^, ^46^, ^47^ the 17 included studies, each based on a unique sample. Countries of origin were the United States, the United Kingdom, Canada, Australia, Israel, and the Netherlands. Sample sizes ranged from 17 to 123 (median 30, total 629). There was no overlap in samples between the studies. Six studies examined populations of women mainly defined by familial risk, 3 examined women with known genetic mutations (all had affected family members), 3 identified women by high scores on multivariate risk estimation tools (again, many women had affected family members), and 2 were studies of BC survivors.

**Table 2:** Summary of included studies

Reference	Country	Sample Characteristic of Interest	Sample	Data Collection Method	Analysis	Aim(s)
Altschuler and Somkin (2005)	USA	Invitees to chemoprevention trial with Gail scores 1.66% or greater.	n = 51 Age: 40‐80+ y Ethnicity: 93.4% White, 2.5% African American, 2.0% Latina, 2.1% other FH: unclear PH: unclear BRCA: not stated	Semistructured interview	Grounded theory	Understand why eligible women at similarly high calculated risk and made different choices about whether or not to join a hormonal therapy trial.
Beesley, Holcombe, Brown, and Salmon (2013)	UK	Women considered for CRRM	n = 60 Age: 24‐68 y Ethnicity: not stated PH: all FH: not stated BRCA: not stated	Case series	Constant comparative	Understand how CRRM decisions are made in practice and identify factors that influence these decisions.
Bennett, Parsons, Brain, and Hood (2010)	UK	High or intermediate risk Claus scores.	n = 30 Age: 48.1 mean (range not stated) Ethnicity: not stated FH: all PH: not stated BRCA: unknown	Semistructured interview	Thematic analysis	Explore factors associated with cancer worry and utilisation of services.
Chalmers and Thomson (1996)	Canada	First‐degree relative with BC.	n = 55 Age: 20‐69 y Ethnicity: not stated FH: all PH: none BRCA: not stated	Semistructured interview	Latent content analysis and constant comparison techniques within symbolic interaction framework.	Describe risk perceptions, identify self‐care needs and practices, identify factors influencing self‐care practices.
Dagan and Goldblatt (2009)	Israel	Asymptomatic BRCA 1/2 carriers	n = 17 Age: 37‐55 y Ethnicity: Israeli Ashkenazi Jewish PH: none FH: all BRCA: all positive	Semistructured interview	Thematic content analysis	Understand lived experiences of asymptomatic BRCA mutation carriers.
Hallowell, Foster, Eeles, Ardern‐Jones, and Watson (2004)	UK	Breast or ovarian cancer survivors who completed genetic testing.	n = 30 Age: 39‐71 y Ethnicity: not stated PH: 27 FH: 26 FDR BRCA: 10 positive, 12 inconclusive, 8 awaiting results	Semistructured interview	Constant comparative	Explore perceptions and experiences of genetic testing and establish information and support needs before and after result.
Heiniger, Butow, Charles, et al. (2015)	Australia	Family histories of BC or ovarian cancer	N = 36 Age: Mean = 46 Ethnicity: mixed PH: not stated FH: all BRCA: 8 positive BRCA1/2 or P53, 8 negative, 20 untested	Semistructured telephone interview	Grounded theory	Explore how risk perceptions are formed In both tested and untested women unaffected by BC but at increased familial risk of breast and/or ovarian cancer and how risk perceptions affect risk management
Hoskins, Roy, and Greene (2012).	USA	Young (age 36 or below) BRCA carriers	n = 60, Age: 21‐36 y Ethnicity: 3% Hispanic, 97% White, 20% Jewish PH: not stated FH: not stated BRCA: all positive	Semistructured telephone interview	Grounded theory	Investigate how, and how much, risk perception become entangled with partner, children, and interpersonal relationship issues.
Howard, Balneaves, Bottodorf & Rodney (2010)	Canada	BRCA carriers	n = 22, Age: 28–80 Ethnicity: diverse—not stated PH: 5 FH: all BRCA: all positive	Semistructured interview	Grounded theory	Understand women's decision‐making processes and the social contexts that influence these processes.
Kelly (1980)	USA	Maternal history of BC	n = 39. Age: 28‐73 y Ethnicity: 34 White, 3 Oriental, 2 Black PH: none FH: all BRCA: not stated	Semistructured interview	Grounded theory	Determine needs, concerns, and health practices pertaining to BC.
Kenen, Ardern‐Jones, and Eeles (2003)	UK	Family history of BC.	n = 21 Age: 24‐61 y Ethnicity: not stated PH: none FH: all BRCA: all unknown	Semistructured interview	Thematic	Report on risk perception, family history, life stages, biographical interruptions, and women's attempts to control their risk
Keogh, McClaren, Apicella, and Hooper (2011)	Australia	1 first or second‐degree relative examined with BC before 50 y. BRCA1/ 2 not identified in family.	n = 24, Age: 35‐70 y Ethnicity: not stated FH: all PH: none BRCA: all unknown	Semistructured interview	Thematic	Explore how women perceive their risk of breast cancer and how this perception influences their screening decisions.
Raveis and Pretter (2005)	USA	Daughters of BC survivors	n = 50. Age: 21‐62 y Ethnicity: 70% White, non‐Hispanic, 24% Hispanic 4% Black, non‐Hispanic. PH: not stated FH: all BRCA: not stated	Semistructured interview	Content analysis	Describe daughter's experience and elucidate reactions to mother's BC diagnosis.
Robertson (2000)	Canada	Breast health clinic attendees, aged 30‐50 (pre‐menopausal); no PH of BC. Only presents data of women indicated to be at increased risk by Gail assessment tool.	n = 20 Age: 30‐50 y Ethnicity: mostly northern European PH: none FH: not stated BRCA: excluded women who had undertaken gene testing	Semistructured interview	Thematic	Explore notion of “phenomenology of risk” to explore women's accounts of their own individual risks for BC.
Sheinfeld Gorin and Albert (2003)	USA	At least one close female relative examined with BC	n = 26. Age Normalizer 49 y, adopter 42 y (mean) Ethnicity: majority white PH: not stated FH: all BRCA: suggest ‘some’ but no %	Semistructured interview	Thematic	Understanding of the effect of risk perception on screening adherence in a woman's natural language
Van Dijk, et al. (2004)	Netherlands	Increased risk attributable to FH	N = 123 Age 47 y (mean) Ethnicity: majority White PH: not stated FH: all BRCA: 9.8%	Semistructured interview	Content analysis with coding for frequency analysis.	Examine how women describe their risk and describe cognitions, emotions, and beliefs associated with risk information in genetic counseling for breast cancer.
Werner‐ Lin (2007)	USA	Young age (22–36) BRCA positive	n = 22, Age: 22–36 Ethnicity: White, mixed Eastern/ Western European PH: none FH: 20 BRCA: all positive	Semistructured interview	Listening guide	Explore connections between family history and beliefs about susceptibility. Understand how family histories/experiences with health care professionals integrate to inform beliefs.

Abbreviation: BC, breast cancer; CRRM, contralateral risk‐reducing mastectomy; FH, family history; FDR, first degree relative.

### 3.1 Overview

Women generally perceived risk categorically rather than probabilistically, partly based on previous family experiences of BC. Decisions about risk reduction arose in 2 very different ways, depending on how intensely women worried about BC.

### 3.2 Women did not perceive risk probabilistically

Only 3 of the 16 studies reported evidence that women viewed risk in likelihood‐value terms. Of these, only Robertson^31^ reported that women made probabilistic risk estimates. In this study, many women spontaneously attempted to create their own personal risk estimate, anchoring this in a known population average and adjusting their estimate according to their personal risk factors. Robertson studied women with elevated Gail risk factor scores but excluded any who had experienced cancer or had been referred for a genetic test. Thus, her sample may have a lower risk profile than other reviewed studies, which might explain her unique finding that women commonly used probabilistic risk perceptions. The other 2 studies reporting probabilistic estimates found these in only a few women. When asked to describe personal risk, only 2 of 24 women in Keogh et al^32^ study of women at familial risk volunteered what could be regarded as continuous estimates (eg, “one in three”). Beesley et al^33^ found, amongst 60 bc survivors requesting contralateral RRM, only 5 reported probabilistic estimates. Indeed, several studies showed that a proportion of women explicitly rejected the notion of trying to understand risk probabilistically or using objective risk estimates.^33^, ^34^

### 3.3 Women perceived risk categorically

Instead, when asked to describe personal risk, most women used verbal labels to describe risk categories to which they felt they belonged.^34^, ^35^, ^36^, ^37^, ^38^, ^39^ Categorisation was pervasive across the differing samples and analysis methods, and women were explicit about doing this. Category labels were diverse. Some, such as “probable,” “high risk,” or certain (of BC) could be seen as ordinal points across a spectrum of likelihood.^34^, ^38^, ^39^ Others described positions relative to population risk, such as “a bit higher than population “risk” or “no higher than anyone else.”^34^, ^36^, ^39^ Other labels encompassed qualitative categories such as “vulnerable” or “at risk.”^39^ Categorisations were generally realistic. Almost all women acknowledged being at high risk in either an absolute sense or comparison with the wider population, and 1 study showed that women's self‐categorisations were largely consistent with the categorical estimates that they had been given by professionals.^38^

Women were explicit about the ways that they developed these categories and about using category labels to help them to think about risk. Some spontaneously assigned themselves to categories, based on either their personal or their family experiences related to cancer (see below), their theories about specific risk factors,^32^, ^34^ or their emotional responses to risk.^34^ Others reported that health professionals introduced them to the categories that they used.^33^, ^39^ In 1 study,^38^ BRCA1/2 mutation carriers wanted health professionals to provide them with risk “labels.”

### 3.4 Family experiences defined risk perceptions

Women's perceived risk categories were informed by family experiences of BC.^34^, ^35^, ^39^ Some women were explicit about how their family histories led them to believe that they were in a high risk category.^39^, ^40^, ^41^ However, in most women, the influence of family experiences was implicit.^33^, ^39^, ^40^, ^41^, ^42^, ^43^ Women assumed that their futures would follow the path of a family member's illness with little consideration that their own cancer likelihoods or experiences might differ.^35^, ^39^, ^40^, ^41^, ^42^ The ages, sites, and stages at which relatives had been examined and the outcomes of relatives' illnesses defined expectations of their own fates, in that they expected to have cancers that would develop with the same trajectories ^[^ 34, 41, 42 ^]^. Relatives' ages were particularly important. Dagan and Goldblatt^41^ referred to this as the “family clock,” Werner‐Lin^40^ as a “danger zone.” Remaining healthy at the age at which their mothers developed BC provided hope for future health,^41^ and women felt profound relief when these landmarks had passed.^34^, ^36^, ^41^ Thus, memories of the experiences themselves constituted risk perceptions by implicitly defining templates of expectations about personal futures. The dominance of family experiences in shaping risk perceptions was not, however, inevitable. Chalmers and Thomson^35^ showed that some women moved away from an experience‐determined view of BC risk toward more nuanced and objectively based understandings over time. These women sought objective information about risk, reflected upon this and integrated it into their views of risk.

Family experiences sometimes led to a sense of inevitability about BC, inducing a “labelling” error whereby BC risk was described as “certain.” This was evident in those whose mothers had been examined with BC^44^ and in some with affected first or second‐degree relatives.^32^, ^35^ After positive BRCA tests, many women in Werner‐Lin^40^ study felt that they were on a “path towards cancer.”

### 3.5 Some women deliberated about risk, but worry impelled others toward aggressive risk‐reduction procedures

Women made decisions in 2 ways, largely depending upon their level of worry. Where worry was not intense, women generally reported weighing their risk of BC and iatrogenic risks associated with risk‐reduction procedures. They also considered other factors, such as how engaging in research might help other women,^34^ how future childbearing and breast‐feeding aspirations might militate against oophorectomy or RRM, and how current parental responsibilities militated against any such major surgery.^35^, ^39^, ^40^, ^41^ Sometimes they postponed decisions if they felt unready to make them.^37^ Unfortunately, the reviewed studies did not reveal how women made comparisons between different risks to make their decisions. Therefore, whilst we know that women largely thought about risk categorically, we do not know how they weighed different risks in reaching decisions. Nonetheless, women who weighed risks and benefits were generally content with their decisions.^37^, ^41^, ^42^, ^43^ Often the decisions provoked strong emotions, but women did not describe these emotions as influencing their decisions.^44^, ^45^

Each study described women whose decisions were shaped by intense levels of worry about BC. Women described fears about BC as ever‐present, intrusive, uncontrollable, and sometimes “intolerable*.*”^31^, ^32^, ^36^, ^41^ The threat of BC was a “constant companion” for these women^31^, ^40^ and induced a persisting sense of threat.^44^ No study explicitly examined why these women worried so intensely, and worry was not clearly explained by the risk category to which women allocated themselves. Whilst some women attributed worry to being in a high risk “category” or to recalling salient family experiences of BC,^33^, ^36^, ^37^, ^38^ women who saw themselves as at high risk did not all worry so intensely.^32^, ^34^ Believing that cancer was inevitable could even reduce worry where women with family history of BC resigned themselves to this.^39^, ^45^

Worry resisted reassurance from professionals' descriptions of objective risk or from good outcomes of clinical investigations.^32^, ^34^, ^37^ Instead, minimising worry became the overriding and urgent goal that women pursued through their decisions about risk reduction.^32^, ^33^, ^36^, ^41^, ^42^, ^43^, ^46^, ^47^ Women acknowledged that they were more worried than their objective risk warranted, but their decisions were nonetheless determined by worry.^33^, ^38^, ^46^ The goal of minimising worry typically led women to eschew conservative options and choose the most aggressive available to them. Worry led women to prefer mammographic screening to self‐care such as breast self‐examination,^46^ participation in a chemoprevention trial to screening,^36^ and RRM to screening.^33^, ^44^ Many described needing to “do something” and feared missing opportunities to reduce risk, but there was little indication that women had closely considered the iatrogenic risks associated with their chosen procedures, particularly RRM or oophorectomy.^31^, ^32^, ^34^, ^42^, ^43^, ^45^

## 4 DISCUSSION

Women did not generally think probabilistically about risk. Instead, they perceived risk in idiographic categorisations, influenced by family experiences. Some women deliberated about their decisions, whereby they weighed risks and benefits of different options. For others, worry excluded deliberation and drove choices of aggressive risk‐reducing options.

Seen from the perspective of research showing that categorisation introduces error into risk‐related health decision making, the central role of categorisation in women's perception of risk is potentially alarming. For example, Cameron et al^48^ showed that the categorisation of probabilistic risk estimates inevitably reduces accuracy by dividing a linear dimension into a restricted set of categories, Thus, miscategorisation can lead to incorrect inferences drawn from category labels. Reyna et al^49^ showed that dependence on categories prevents people from acting upon category‐inconsistent information. In our review, 1 potentially major source of error in categorical risk perceptions was that women inferred their risk category from memories of family experiences. Many categorised risk as certain or saw themselves on an inevitable “path toward cancer.” These perceptions arose inductively, and women did not objectively assess the significance of family experiences. Family BC history does, in general, increase personal risk,^6^ but valid inferences should take account of the number of affected relatives and their ages and their genetic relationship to the individual. Women in the studies that we reviewed were influenced mainly by aspects of the familial experience unrelated to risk, such as the quality of family relationships and specific details of relatives' experiences.

However, wider research in social psychology shows categorisation to be a fundamental aspect of how people interpret information and use it to make decisions.^50^, ^51^ Categorisation is a heuristic that allows people to remember, retrieve, and use risk information more easily.^52^ Thus, category labels such as “above average” or “at risk” provided easily accessible meanings that could help women to make decisions. Although categorisation of risk could be biased by family history, women wanted to use accurate categories. In particular, they sought accurate categorical information from health professionals^43^ and were able to remember these when later asked.^38^ Therefore, categorisation should not be regarded as an inherently inaccurate way for women to perceive risk.

Unfortunately, the reviewed studies did not illuminate how women compared different risks to make their decisions. Unlike probabilistic risk perceptions, where a common metric allows comparison of different outcome probabilities, categories used by women in the reviewed studies carry unique meanings that do not offer a common metric.^48^ For example, patients considering surgery may use different types of category to describe risk of BC (eg, “I am vulnerable”) and surgery (eg, “I am at moderate risk”). One possibility suggested by the broader psychological literature is that some risk categories, such as feeling “vulnerable,” carry stronger emotional connotations than others, such as having “moderate” risk. The risk associated with the stronger emotional connotation might therefore drive the decision.^27^ Research is needed to elucidate how women compare categorical risk perceptions in decisions about risk reduction.

The clearest evidence about decision making in the reviewed papers arose where it was influenced by worry. Where worry was intense, women did not consider risks and benefits. Instead, they chose aggressive risk reduction to reduce worry, and they paid little attention to iatrogenic risk. That is, worry “hijacked” decision making. At first sight, this is consistent with evidence for widespread use of a heuristic whereby people infer risk from their emotional responses.^53^, ^54^ However, our review emphasised another mechanism: that worry reduction became a decision‐making goal in its own right. That is, women pursued an “emotion‐focussed” coping strategy where they sought to reduce worry and were largely unconcerned with objective risk.^54^, ^55^

### 4.1 Practice and research implications

Our findings point to 2 challenges for practitioners working with women who are deciding about BC risk reduction. First, women's categorical perceptions of risk may not closely correspond to objective risk, and second, worry might hijack their decision making by leading women to choose invasive procedures to reduce worry whilst disregarding objective risks and benefits.

Risk perception research shows that categorisations and other heuristics that people use to perceive risk are not static but evolve toward greater nuance and accuracy if people engage with external evidence.^24^ Our review shows that women sought accuracy in their categorisations and formed more nuanced categories over time by seeking information about the categories to which they belonged from health professionals.^38^ Relevant information does not necessarily have to be presented didactically, and indeed, didactic presentations may be counterproductive.^56^ Street^56^ and Elwyn et al^57^ both emphasise the centrality of patient‐clinician dialogue in enhancing the extent to which patients are able to make well‐informed decisions based on risks and benefits; it allows clinicians to assess patients' understanding and tailor information to patients' needs, and patients to test and improve their understanding based on clinicians' feedback. Thus, effective consultation with women considering risk reduction does not merely extend to providing information about risk but involves clinicians eliciting and shaping women's own idiographic categorisations of risk.

The influence of worry is harder to address. Where it is linked to perceiving oneself to be at high risk of BC, worry might be alleviated by helping women to question the risk perceptions that they have formed, as we describe above. However, cancer worry is not simply a product of high‐risk perceptions and indeed appears to be largely insensitive to reassurance about risk.^54^ It is partly a consequence of unrelated factors, including general emotional state, negative life experiences, and stressful or unsupportive environments.^58^ Whilst counseling or other interventions for worry might therefore prove helpful in some instances, it is unrealistic to expect that worry can be completely banished in clinical contexts in which patients are faced with the mortal threat of serious illness. Therefore, particularly when the clinical benefits of interventions are unclear, clinicians will face the dilemma of how to reconcile the normative expectations for them to respect patients' requests, motivated by escape from worry, whilst simultaneously meeting their needs, which go beyond worry to encompass the balance between reduction of BC risk and avoidance of iatrogenic harm. The dilemma hinges on the ethical question as to whether and when it is appropriate for clinicians to provide invasive, and even surgical, responses to psychological need. Ethical analyses in the fields of cosmetic^59^ and bariatric surgery^60^ suggest surgical intervention for psychological benefit can be justified, but that a case always needs to be made that clinical and psychological benefits outweigh risk, that benefits are likely to occur and that benefits cannot be achieved with less risk.

Where solutions to ethical dilemmas cannot be derived from general normative principles, Kleinman^61^ proposed that the starting point for reflecting on possible solutions should be detailed study of how practitioners and patients resolve these dilemmas in practice. Unfortunately, the studies that we reviewed provided little insight into this. Future research that examines, not only women's accounts but also those of the clinicians caring for them, and which examines how decisions are negotiated between them in consultations, could provide evidence from which realistic and ethically robust solutions can be derived.
